# Development of a DNA Barcoding System for Seagrasses: Successful but Not Simple

**DOI:** 10.1371/journal.pone.0029987

**Published:** 2012-01-11

**Authors:** Christina Lucas, Thirunavakkarasu Thangaradjou, Jutta Papenbrock

**Affiliations:** 1 Institute for Botany, Leibniz University Hannover, Hannover, Lower Saxony, Germany; 2 Centre of Advanced Study in Marine Biology, Annamalai University, Parangipettai, Tamilnadu, India; American Museum of Natural History, United States of America

## Abstract

Seagrasses, a unique group of submerged flowering plants, profoundly influence the physical, chemical and biological environments of coastal waters through their high primary productivity and nutrient recycling ability. They provide habitat for aquatic life, alter water flow, stabilize the ground and mitigate the impact of nutrient pollution. at the coast region. Although on a global scale seagrasses represent less than 0.1% of the angiosperm taxa, the taxonomical ambiguity in delineating seagrass species is high. Thus, the taxonomy of several genera is unsolved. While seagrasses are capable of performing both, sexual and asexual reproduction, vegetative reproduction is common and sexual progenies are always short lived and epimeral in nature. This makes species differentiation often difficult, especially for non-taxonomists since the flower as a distinct morphological trait is missing. Our goal is to develop a DNA barcoding system assisting also non-taxonomists to identify regional seagrass species. The results will be corroborated by publicly available sequence data. The main focus is on the 14 described seagrass species of India, supplemented with seagrasses from temperate regions. According to the recommendations of the Consortium for the Barcoding of Life (CBOL) *rbcL* and *matK* were used in this study. After optimization of the DNA extraction method from preserved seagrass material, the respective sequences were amplified from all species analyzed. Tree- and character-based approaches demonstrate that the *rbcL* sequence fragment is capable of resolving up to family and genus level. Only *matK* sequences were reliable in resolving species and partially the ecotype level. Additionally, a plastidic gene spacer was included in the analysis to confirm the identification level. Although the analysis of these three loci solved several nodes, a few complexes remained unsolved, even when constructing a combined tree for all three loci. Our approaches contribute to the understanding of the morphological plasticity of seagrasses versus genetic differentiation.

## Introduction

Seagrasses are higher plants capable to complete their life cycle under submerged conditions in the marine environment [1]. They evolved independently at least three times between 75 and 17 million years ago [2]–[4]; hence, seagrasses form a paraphyletic group including four core families (Cymodoceaceae, Hydrocharitaceae, Posidoniaceae, and Zosteraceae). These marine plants cover large geographic ranges worldwide [5], surviving most diverse environmental conditions. They have fundamental roles in the ecology of coastal areas, e.g. as breeding and nursery ground for a variety of marine organisms, coastal stabilizers or coast protectors next to coral reefs and mangroves. Decline in seagrass species and cover were observed throughout the world and the recent estimates indicate that these resources are gradually disappearing at the rate of 110 km^2^ yr^−1^ since 1980 [6]. Main factors for the loss of seagrasses are eutrophication and high turbidity due to natural and human influences. Furthermore seagrasses contain highly valuable secondary compounds such as phenolic acids used for traditional medicine and biotechnological purposes (e.g. rosmarinic acid as antioxidant or zosteric acid as an effective antifouling agent).

Due to the similar lifestyle, morphology of seagrasses is reduced and shares a number of similarities e.g. strap-like leaves in case of Posidoniaceae, Cymododoceaceae and Zosteraceae. In addition, seagrasses predominantly propagate by vegetative growth in units, so called ramets. Sexual reproduction occurs rarely due to irregular and seldom flowering [7]. Another phenomenon is the ‘trait’ of morphological plasticity within the same species, hypothesized to enable survival in different ecological niches [8]. All these factors make correct assignment of species based alone on conventional identification keys difficult or even impossible. For these reasons, closely related species e.g. *Halophila* ssp. [9] still form a taxonomically unresolved complex without correct assignment of species and subspecies. Even molecular phylogenies using *matK*, *rbcL*, and *trnK* and ITS spacer regions yielded in different results resolving the genus complexes. Moreover, only Australian, Southeast Asian and Mediterranean species are included in the molecular databases. At present, no molecular data exist to elucidate Indian seagrass communities.

The accelerating decrease of seagrass meadows in India and other parts of the world, as well as lack of traditional conservation approaches (e.g. cultivation in botanical gardens) were the main reasons to invest in a seagrass barcoding system.

DNA barcoding is defined as: Methods for identifying species by using short orthologous DNA sequences, known as “DNA barcodes”, that have been proposed and initiated to facilitate biodiversity studies, identify juveniles, associate sexes, and enhance forensic analyses [10], [11]. The criteria for the development of reliable barcode data was defined by the Consortium for the Barcoding of Life (CBOL) as follows: Candidate loci should be suitable for a wide range of taxa, show a high variation between species, but should be conserved within species, so that the intra-specific variation will be insignificant [12]. Ideal barcodes should be routinely retrievable with a single primer pair, be amenable to bidirectional sequencing with little requirement for manual editing of sequence traces and be short enough to ease PCR amplification. Alignment and analysis of the resulting sequences ought to be straightforward, allowing a fast identification without profound prior knowledge about bioinformatics.

Short standard regions that enable cost-effective species identification are preferable, such as cytochrome C oxidase (COI) for most animal species [10], [13]. Within 2 h the express barcode system can be successfully recognize and identify certain animal species [14], coming close to the goal of fast and cheap identification. However, so far there is no generally accepted DNA barcode standard for the plant kingdom although a number of recommendations by the CBOL plant working group [12] and others [15]–[20] exists. The performance of different loci combinations remains insufficient among different plant families [18], [21], [22]. Some researchers solved it by designing family specific primers and came closer to accepted phylogeny using this approach [23].

Sequences in the plant chondriom evolve too slowly; therefore they cannot be used for discrimination. The nuclear genes contain introns and frequent recombination is observed. Prior knowledge about copy number and linkage groups is needed to perform a neutral analysis [24]. Therefore genes from the plastid genome are the most promising candidate genes for plant DNA barcoding. The plastid genome is uniparentally inherited, non-recombining, and a structurally stable genome [11]. Plastid regions with raw sequence differences ≥2% were categorized as the most variable segments, and therefore the most promising of the plastid genome for DNA barcoding when normalized for length of 300 to 800 bp. Current results indicate that at least two plastid genes, better a multi-locus code, are necessary to specify a reliable plant DNA barcode, one as a “robust and basic” gene like *rbcL* and one a more “variable” like an internal spacer (e.g. *rps16*, *trnH-psbA*) or *matK*. Resulting barcodes of the “robust” gene *rbcL* could be identified using BLAST and resolve to genus and family level, while the “variable” part of the code is for species verification. So far, most promising combinations are *rbcL/matK* [12] and *rbcL/psbA-trnH* [11]. Single-locus barcodes for plants are not advisable, although proposed for *matK* [16], [25] but clearly lacking the feature of universal amplification. In this approach, we followed the CBOL recommendations and expanded the two-locus system by incorporation of the *trnH-psbA* spacer as a third locus. This spacer region tends to be more variable and might enhance discrimination power of the two-locus system.

Besides identification of suitable DNA barcoding sequences, reliable searching algorithm and specialized barcode databases are currently missing [26]. So far, barcoding is relying on methods used in phylogeny although goals are different: (1) fast identification of an operating taxonomic unit, (2) screening of diversity hot spots (3) and no compulsory resolution of relationships across complete families including hybrids and introgression.

It is well known that tools for tree-based analysis methods have several disadvantages concerning their application on DNA barcoding [27]. Therefore, a character-based method such as the Character Attribute Organization System (CAOS, reviewed in [28]) is a promising approach to combine all kinds of data sets goaling at a comprehensive barcoding platform. Additional to these methods, verification of the species using BLAST algorithm based on the data deposited on NCBI was tried. Up to date the CBOL database is only supporting search with *matK* and *rbcL* as barcodes, based on a small dataset which was not supporting seagrasses or related plant families.

Seagrasses comprise a limited number of species. Several genera are monospecific or show small species representation and their genetic plasticity is limited. Moreover hydrophilic pollination limits the gene flow across the water bodies [29]. On the contrary, recent comparisons of ITS sequences from Chinese and Australian material of *Enhalus*, a monospecific genus, show that they are quite distinct. This warrants the possibility of the existence of additional new species [6]. Though different markers were used for different species (*rbcL* and *matK* of the Hydrocharitaceae [30], nuclear (18SrRNA, ITS) and other cpDNA (*trnL*) loci in *Thalassia*, nuclear (ITS) and cpDNA (*rbcL*, *trnL*) in *Halodule* [5], chloroplast *trnL* intron and *rbcL* of several seagrasses [31], nuclear (ITS) and cpDNA (*rbcL*, *trnL*) loci of *Posidonia*, nuclear ITS of *Halophila* [32], ITS (ITS-1 and ITS-2 regions including the 5.8S rDNA gene), *trnK* introns and *rbcL* of *Zostera* [33], *rbcL* and *matK* of *Zostera* [4], ITS1, 5.8S rDNA and ITS2 of *Halophila* [34], and ITS1, 5.8S rDNA and ITS2 of *Halophila* [35]) there is no generally agreed consensus on conserved molecular region useful for seagrass taxonomy.

The International Union for Conservation of Nature provides a criterion giving equal importance to conserve both: genetic diversity and ecosystem diversity. Spielman et al. (2004) [36] emphasized that most species are not driven to extinction before genetic factors impact them and thus ecological, demographic and genetic processes must be considered together. Therefore a clear understanding of genetic diversity of the seagrass species becomes important by considering the global seagrass decline. But Jones et al. (2008) [37] alerted that the genetic similarity was highest within each of the seagrass beds indicating a low degree of gene flow from one population to another at different sites and suggested that future restoration and conservation projects should use only local eco-sourced materials rather than using populations of different regions.

Despite several studies on molecular taxonomy of seagrasses, there is no seagrass sequence in the CBOL database. Therefore, a barcoding system for seagrasses has to be developed based on the experiences with related species. Hence the present study was carried out with the aim to develop a simple but efficient DNA barcoding system for seagrasses, especially for sampling in the tropics. The primer universality for seagrasses was tested. The number of loci for species discrimination and the percentage that can be discriminated was determined by analysing the combination of different loci.

## Materials and Methods

### 2. 1 Plant Material

Plant material from tropical seagrasses [*Cymodocea rotundata* Ehrenb. & Hempr. ex Asch., *Cymodocea serrulata* (R.Br.) Asch. & Magnus, *Enhalus acoroides* (L. f.) Royle, *Halodule pinifolia* (Miki) den Hartog, *Halodule uninervis* (Forsk.) Asch., *Halodule wrightii* Asch., *Halophila beccarii* Asch., *Halophila decipiens* Ostenf., *Halophila ovalis* (R.Br.) Hook. f., *Halophila ovata* Gaud., *Halophila ovalis* subsp. *ramamurthiana*, *Halophila stipulacea* (Forsk.) Asch., *Syringodium isoetifolium* (Asch.) Dandy, *Thalassia hemprichii* (Ehrenb.) Asch.] were collected in the Palk Bay, Tamil Nadu, and in the Chilika Lagoon, Orissa, India while plant material of temperate seagrasses was collected in three different locations on the North sea island Sylt: *Zostera marina* L. (three populations), *Zostera noltii* L. (two populations). The longitude and latitude coordinates were determined by using google maps location (http://www.gorissen.info/Pierre/maps/googleMapLocationv3.php) (Table S1).

### 2.2 DNA extraction

Several methods including shade-drying, immersion in high NaCl/cetyltrimethylammonium bromide (CTAB) buffer or freezing in liquid nitrogen were applied to preserve the plant material for DNA extraction. In general, the plants were dug out and cleaned in seawater to remove debris and epiphytes. Clean seagrass leaf samples were directly submerged in CTAB buffer [38]. Others were dried overnight at a dry and dark place. The material was grinded to a fine powder at 22 Hz for 2 min using a bead mill (Retsch, Haan, Germany). Then the material was stored at −80°C until analysis. The DNA was extracted from 5 to 10 mg plant material by using the Nucleo Spin Plant Kit II (Machery & Nagel, Düren, Germany) with slight modifications. The first step, incubation time in CTAB buffer, was prolonged to 30 min. The concentration was determined spectrophotometrically (Synergy MX, BioTek Instruments, Bad Friedrichshall, Germany) and analyzed for its intactness by agarose gel electrophoresis.

### 2.3 PCR conditions

Amplification of *rbcL* and *matK* sequences by PCR was performed using conditions suggested by the Plant working group of the CBOL [12]. The following primer pairs were designed: P609 5′-GTAAAATCAAGTCCACCRCG-3′ and P610 5′-ATGTCACCACAAACAGAGACTAAAGC-3′ [17] for a *rbcL* fragment of 599 bp for all species; P607 5′-CGTACAGTACTTTTGTGTTTACGAG-3′ and P608 5′-ACCCAGTCCATCTGGAAATCTTGGTTC-3′ for a *matK* fragment of 889 bp for *C. rotundata*, *C. serulata*, *H. ovata*, *Z. marina* sp., *Z. noltii* sp. (Ki-Joong Kim, Korea, unpublished), and P646 5′-TAATTTACGATCAATTCATTC-3′ and P647 5′-GTTCTAGCACAAGAAAGTCG-3′ [39] for a *matK* of 945 bp for *E. acoroides*, *H. becarrii*, *H. decipiens*, *H. ovalis*, *H. pinifolia*, *H. uninervis*, *S. isoetifolium*, *T. hemprichii* (Palk Bay) and *H. pinifolia*, *H. uninervis*, *H. beccarii*, *H. ovalis* (Chilika Lagoon).

The PCR analysis was extended using the following primer pairs for all species: P672 5′-GCGTGGCCAAGYGGTAAGGC-3′ (trnQ^(UUG)^) and P673 5′-GTTGCTTTYTACCACATCGTTT-3′ (rpS16x1) for the amplification of 850 and 1,400 bp [20], P674 5′-CCTTATCATTTAGAGGAAGGAG-3′ (ITS5a) [40], [11] and P675 5′-TCCTCCGCTTATTGATATGC-3′ (ITS4) [41] for ITS of 900 bp and 1,800 bp, P676 5′-GTTATGCATGAACGTAATGCTC-3′ (psbA3′f) [17], [42] and P677 5′-CGCGCATGGTGGATTCACAATCC-3′ (trnHf) [43] *for the trnH-psbA spacer of 296 to 415 bp*.

The following PCR conditions using Dream Taq polymerase (MBI Fermentas, St. Leon-Rot, Germany) with P609/P610 were applied: 1× Dream Taq Green buffer, 0.2 mM dNTPs, 1% BSA, 1% PVP, 2 mM MgCl_2_, 1 U Taq polymerase, 10 to 30 ng template DNA, 1 pmol primer each in a total volume of 25 µl. The PCR was performed in a PTC 200 thermocycler (BiozymDiagnostik GmbH, Hess. Oldendorf, Germany) with a heated lid under the following conditions: an initial denaturation (95°C, 5 min) followed by 30 cycles with a denaturation of 94°C for 30 s, an annealing of 56°C for 35 s, an extension of 70°C for 60 s, and a final extension of 72°C for 8 min. PVP was added to bind and precipitate the large amounts of phenolic acids in DNA preparations of seagrasses.

With P607/P608 following conditions were applied: 1.5× Dream Taq Green buffer, 0.2 mM dNTPs, 1% BSA, 2% DMSO, 1% PVP, 3 mM MgCl_2_, 1 U Taq polymerase, 20 to 50 ng template DNA, 1.5 pmol primer each in a total volume of 25 µl. The PCR was performed under the following conditions: After a pre-cycling of five cycles (initial denaturation, 95°C, 5 min) followed with a denaturation of 95°C for 60 s, an annealing of 49°C for 80 s, an extension of 70°C for 120 s the full cycling of 30 cycles with a denaturation of 95°C for 30 s, an annealing of 56°C for 45 s, an extension of 70°C for 80 s, and a final extension of 70°C for 8 min.

With P646/P647 following conditions were applied: 1× Dream Taq Green buffer, 0.2 mM dNTPs, 1% BSA, 4% DMSO, 1 U Taq polymerase, 20 to50 ng template DNA, 1 pmol primer each in a total volume of 25 µl. The PCR was performed under the following conditions: an initial denaturation (95°C, 3 min) followed by 30 cycles with a denaturation of 95°C for 30 s, an annealing of 50°C for 40 s, an extension of 72°C for 40 s, and a final extension of 72°C for 5 min. For the other primer pairs, cycling conditions where adapted as already published [11], [20].

All PCR reactions were repeated three times independently to reduce errors in the final consensus sequence to a minimum. The PCR fragments were eluted (GenElute, Sigma Aldrich, Taufkirchen) and ligated into the pGEM-T vector (Promega, Mannheim, Germany). Colonies were analyzed by colony PCR and the clones by restriction analysis. Sequencing was done by Eurofins MWG Operon (Ebersberg, Germany) and GATC (Konstanz, Germany) from both directions resulting in a six times coverage for all sequences analyzed.

### 2.4 Bioinformatic analysis

#### 2.4.1 Assembly of obtained sequences

The obtained raw sequence data was analyzed using Clone Manager 9 (Sci-Ed, Cary, NC, USA). The sequence files obtained were assembled and analysed by choosing “Simple”, a method for optimized small sequence sets. Parameters were left at default [Expected coverage = 6x, Overlap score = 50]. The sequence assembly was manually edited to obtain a consensus sequence. The consensus sequence was subsequently analyzed using BLAST [nucleotide blast; database: Others nr; BLAST algorithm: Megablast; Algorithm parameters: default] to verify the gene fragment and/or taxon. After verification, the sequence was examined for the appropriate forward and reverse primer sequences. Sequences flanked by the specific primers were maintained while the contaminating vector sequence was discarded. The resulting sequences were subsequently used for phylogenetic analysis.

#### 2.4.2 Analysis and sequence alignment

For sequence alignment and creation of the NEXUS file, Bioedit 7.0.9 [44] was used. The sequence alignment was carried out with the implemented CLUSTAL X using default parameters. The alignment was further optimized manually and exported as NEXUS file format to identify an evolutionary model by jModel Test 0.1.1 [45], for phylogenetic analysis using MrBayes [46] and for the CAOS-workbench [47]. All sites were included into the analysis, especially in the case of *trnH-psbA*.

#### 2.4.3 Character-based analysis

The search for diagnostic characters was performed with the web-based CAOS Workbench (http://www.cs.ubc.ca/~tmm/papers/tj/; [47]). For this, the aligned sequences and the tree from ML-analysis were loaded in the workbench. To search for diagnostic characters, CAOS-Analyzer and CAOS-Barcoder were used. For the seagrass dataset only the *matK* and *rbcL* marker were chosen, as the alignment of the plastid spacer *trnH-psbA* caused several problems. These two sets were analyzed independently. The software does not provide a continuous analysis so far.

#### 2.4.4 Tree-based analysis

For comparative analysis MEGA 5.0 was used, supporting phylogenetic tree reconstruction methods such as Neighbour-Joining (NJ), Maximum Likelihood (ML) and Maximum Parsimony (MP) [48]. The distribution of sites in the dataset was computed also by using MEGA 5.0. Combination matrices of different loci were obtained using SequenceMatrix 1.6.5 [49]. For phylogenetics, the optimal fitting evolutionary model was determined by jModel Test 0.1.1. This is a commonly used program, carrying out different calculations to identify the best fitting model dependent on sequence length, sample size, and purpose of analysis.

The NEXUS file alignment was loaded into the software and likelihood scores for 24 substitution models were computed [Number of substitution schemes: 3, Base frequencies: +F, Rate variation: +G +I nCat = 4, Base tree = ML optimized]. After computation of the likelihood scores by the implemented PHYML algorithm [50], different statistical calculations were carried out: Akaike Information Criterion [parameters: default, small-sample-size correction (AICc)], Bayesian Information Criterion [parameters: default] and Decision Theory [parameters: default] test. Results were compared and the consensus optimal model was implemented in the phylogenetic software with likelihood computations suggested by PHYML. The appropriate and supported model was then used for ML and Bayesian Analysis. The following model parameters were used for NJ and MP analysis: *rbcL*, *rbcL/matK*, *trnH-psbA/rbcL* and *matK/trnH-psbA/rbcL* GTR+G and for *matK*, *trnH-psbA*, and *matK/trnH-psbA* GTR+I+G.

For each analysis in MrBayes the following parameters were used: mcmcp ngen = 1,000,000, nruns = 2, nchains = 4, temp = 0.100, printfreq = 1,000, samplefreq = 100, diagnfreq = 100, stop rule = yes, stopval = 0.01, burninfrac = 0.33, nswaps = 3. No heating was applied based on preliminary results (data not shown). A stop rule was programmed, stopping the analysis when standard deviation of split frequencies was below 0.01. When more than one locus was used, the matrix was set up as portioned one, allowing an unlinked analysis. According to this setup analysis was stopped when 33% of the samples were deleted.

For ML the same model was used, considering a bootstrap value of 1,000 and Nearest-Neighbour-Interchange. The analysis of MP and NJ was performed using *p*-distance as model and pairwise deletion for gap treatment, also considering a bootstrap value of 1,000. The resulting consensus trees were analyzed for branch support and topology congruence. Mean support and standard deviation for each method and each locus was determined for comparison. For the combined tree, trees of single- and double-loci analysis were combined with the help of TreeJuxtaposer [51], generating a consensus topology of all methods and loci-combinations.

#### 2.4.5 Distance analysis

For the distance analysis, Species Identifier was used in a modified way. Since sampling strategy did not support species identification on a statistical reliable basis, genus level was used as minimal taxonomical unit. Following this approach the intra- and inter genus distances were determined. Mean overall, intra- and inter species distances were calculated as well as the distribution of variance among the different loci.

#### 2.4.6 Inverted repeat (IR) analysis

Analysis of the IR regions of the *trnH-psbA* spacer, was performed as recently reported by Whitlock et al. (2010) [52], using EMBOSS Software package [53]. The EINVERTED algorithm was used with a modified threshold of 40 to analyze the short spacer fragment properly.

## Results

After optimization of the DNA extraction method from seagrasses, amplicons for almost all species and primer pairs could be produced in the PCR reactions. Especially for dried herbarium samples the extracted DNA was often degraded and the amplification of large fragments difficult. The recommended primer pairs for the amplification of *rbcL* [12] worked well in case of *rbcL* after PCR optimization. For the *matK locus*, another primer pair as recommended by Ford et al. (2009) [39] lead to successful and reproducible amplification although the primers deviate only slightly. In trials with the CBOL recommended pair reverse-reverse primer 840 bp-fragments were observed in some seagrass species. The best working combination was primer pair 608 and 647 at a wide range of annealing temperatures. The primer universality is summarized in Table S2. For the larger fragments - *matK* and *rbcL* - sequence identity was verified by bidirectional sequencing, with less than 0.01% error rates. For the short *trnH-psbA* spacer region bidirectional sequencing was always accurate. Consequently, *trnH-psbA* was only sequenced in one direction for the rest of the analysis.

Since BLAST can be used as a rapid identification tool [10], its performance was tested on the seagrass sequences. Over 80% of the taxa used in this study were deposited on NCBI taxonomy. In 62% of all taxa the *rbcL* sequence could be used for direct comparison, but less for *matK* and *trnH-psbA* (37% each). Sequence overlap was generally close to 99%. Only in the case of *H. uninervis* the support was lowered to 86% (Table S1, S3). The BLAST identification level was analyzed for all species, taking the first five BLAST hits into account. Based on this, nearly all species could be identified to genus level. For these with a corresponding sequence often species level was reached.

The complete sequence sets of *rbcL* and *matK* were analyzed first by tree-based methods focussing on resolution and topology. Exemplary trees obtained by NJ analysis (1,000 Bootstrap replicates) of both sequences were constructed and compared using TreeJuxtaposer [51] (Figure S1). The species resolution and topology analysis underlines the need of a two-locus barcode, since species identification was not reliable using a single-locus system. The lines and taxa indicated in red (Figure S1) reveal different topologies among the trees indicating that both sequence sets reflect different relationships. The resolution is not satisfying, in particular for the *Halophila* complex and the *Halodule* species. The other algorithms, ML, MP, and Bayesian analysis, used for tree generation resulted in similar conclusions. The DNA barcoding would be of highest benefit to distinguish between the *Halophila* species because of their morphological similarity.

**Figure 1:**
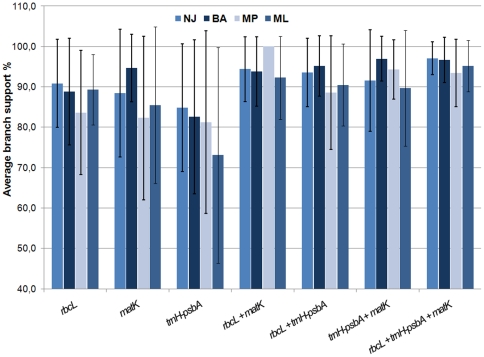
Average branch support analysis across different loci and phylogenetic methods. Data of single- and combined-loci was analyzed using Bayesian analysis (Bayes), Maximum Likelihood (ML), Neighbor Joining (NJ) and Maximum Parsimony (MP). The average branch support was calculated for well resolved clades (above 50%), resulting from all methods used. Error bars indicate standard error of support values. The average support varied significantly using different methods for single locus analysis, but decreased with the combination of loci.

**Figure 2:**
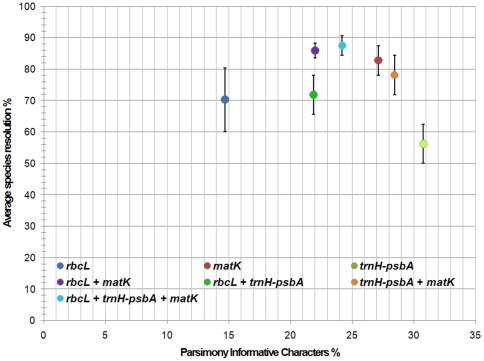
Higher content of parsimony informative characters (PIC) is not related to high species resolution. The percentage of PIC was plotted across the average species resolution calculated over all methods used for single or combined datasets. Error bars show standard error of species resolution among different methods used. *trnH-psbA* spacer performed poorly compared to *matK* and *rbcL* in single locus analysis, while *rbcL/matK* nearly reach the same resolution percentage as the three-loci combination.

Testing different combinations of two-loci barcodes revealed higher resolution as well as decreased statistical errors when comparing the different algorithms (Figure 1 and 2). The resolution obtained by distance-based methods like NJ and MP was always higher compared to phylogeny model-based algorithms like Bayesian analysis and ML. In the beginning of the project species resolution obtained by a two-locus barcode was poor, resulting in the need of a third locus. However, during the project the number of sampled taxa was increased and higher number of samples also improved results obtained by the two-locus barcode. Moreover *matK* amplification was also tested on herbarium samples, ranging from 100- to five-year-old material, without any satisfying result. Since shortness and high variability (e.g. *trnH-psbA*, Figure S2) are crucial parameters a spacer region was chosen for further analysis. Based on literature [13], [54] it was decided to test three spacer fragments from the plastid and the nuclear genome: *trnH-psbA*, *rps16-trnQ* and ITS. All three loci showed 100% universality for seagrass (Table S2).

Gel-based analysis of *trnH-psbA* amplicons showed an optimal result concerning length and its variability. Furthermore this spacer was successfully included in several hot spot barcoding projects [55], [56]. ITS was less variable focused on length, and *rps16-trnQ* resulted in sizes inapplicable for barcoding purposes.

Since the number of parsimony informative characters (PIC) in a sequence set is one of the basic parameters, the whole dataset and combinations of loci were analyzed for PICs (Figure S2). As expected from other barcoding studies, *rbcL* showed the highest content of conserved sites, the *trnH-psbA* spacer the lowest and the opposite in the case of variable characters. The percentage of singleton sites was highest in *rbcL* which could be useful in a character-based approach. Combination of the loci revealed an average content of about 24% PIC. The most promising combination, focused on PIC content is *trnH*-*psbA/matK* with 28.4% PIC, while *matK/rbcL* and *rbcL/trnH-psbA* did poorly with only 22% PIC (Figure S2).

**Figure 3:**
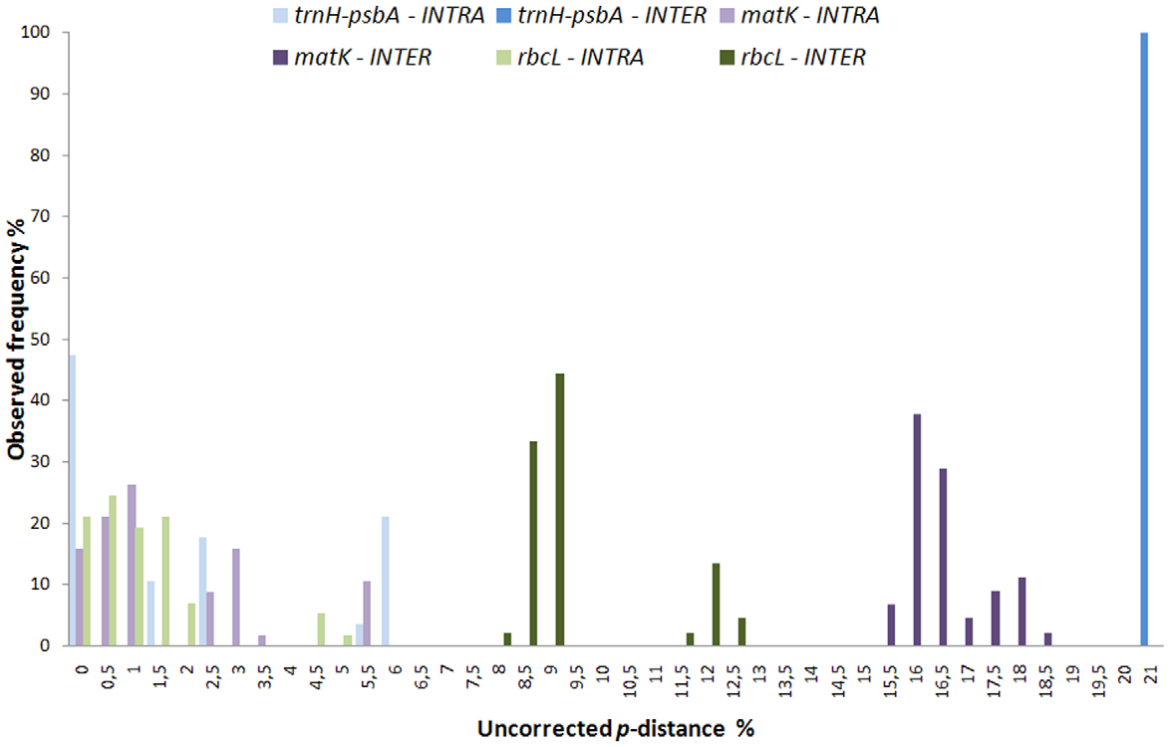
Observed frequency of uncorrected intra- and intergeneric *p*-distance. The frequency of the uncorrected *p*-distance was analyzed to visualize the “barcoding gap”. Frequency was logged on a scale from 0 to 20% distance, indicating that *trnH-psbA* is out performing with an average inter-specific *p*-distance of more than 20%. Inter-generic *p-*distance shown in darker color and intra-generic *p*-distance lighter color, blue for *trnH-psbA* spacer, violet for *matK* and green for *rbcL*, respectively.

We expected that a high percentage of PIC and high singleton content would be coupled with a high species discrimination power. Therefore, the percentage of PIC was compared over the species resolution for the different loci and combinations (Figure 2). The all-loci combination and *rbcL/matK* were out-performing the single combinations and all other combinations. Although *trnH-psbA* showed the highest variability (>30%), it resulted in lowest discrimination of seagrass species (<60%). Therefore, we analyzed the “barcoding gap” for all loci used. In particular for seagrass species which show a high phenotypic plasticity the continuous discrimination power between species and genus is a parameter of high importance. The frequency of uncorrected intra- and inter-generic *p*-distance was analyzed to visualize the “barcoding gap” (Figure 3). The data clearly indicated that *trnH*-*psbA* is out-performing with an average inter-generic distance of more than 20%. The intra-generic distance was observed less than 6%, in most cases (47%) even less than 0.5% distance.

An optimal locus should show a continuous distribution [57]. *rbcL* as single locus is closest to this optimum, *matK* was useful but performed better in combination with *rbcL*. The intra-specific distance could not be determined due to the sampling strategy. Therefore the intra-generic distance was used, pointing out that the *p*-distance performance of *trnH-psbA* is not appropriate for inclusion in a robust barcode system for seagrass.

The alignment of the spacer at the 3′ end was almost impossible whereas the 5′ prime end was usable for further analysis. The species resolution did not differ when excluding the 3′ end in the alignment-based analysis of the dataset. Since it is known that this spacer often contains an inverted repeat (IR) region [52] analysis was performed (Figure S3) with EINVERTED [50]. Interestingly, *Halodule* sp. and *Halophila* sp. shared with only little exchange the IR sequence with *Cymodocea*, *Thalassia* and *Enhalus* as well. Only *Zostera* possessed a differently located IR, supporting divergent evolution. Conservation is visible in a new IR close to the 3′ end which was also partially found in *Syringodium*.

Since there is no common barcoding and visualization software available, several tree- and character-based approaches were used. The data of single- and combined-loci were analyzed using Bayesian analysis, ML, NJ, and MP. The average branch support was calculated for well resolved clades (above 50%), resulting from all used methods. The average support varied significantly using different methods for single-locus analysis, and increased with the combination of loci.

**Figure 4:**
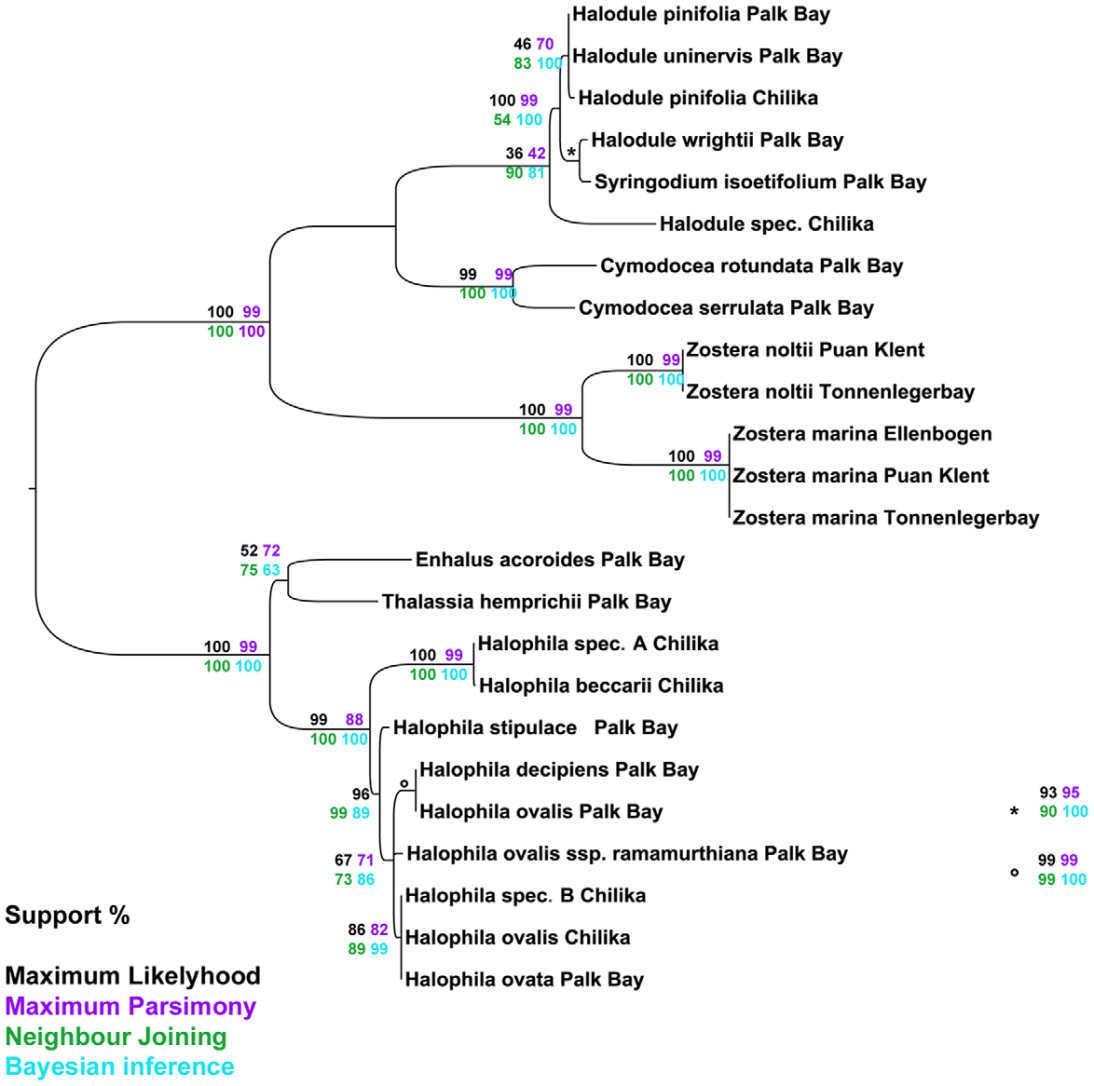
Meta tree of all taxa using *matK/rbcL*. Combined tree of *matK/rbcL* loci, branch support values are given in percent. Support values highlighted by the method used: Maximum Likelihood (black), Maximum Parsimony (violet), Neighbor Joining (green) and Bayesian Analysis (turquoise). Taxa that differed in topology along the different methods are shaded in violet.

The combination of *rbcL/matK* resulted in 100% average branch support, without any ambiguity in MP algorithm. On average the third locus increased the branch support by about 5% (Figure 1). The inclusion of *trnH-psbA* in *rbcL* increased the species resolving power of *rbcL* less than 2% in comparison to *rbcL* alone. By combination of *trnH-psbA* with *matK* the species resolving power of *matK* decreased from 82% to 79%. Here it is evident that *trnH-psbA* when combined with *rbcL* or *matK* lowered the average branch support irrespective of algorithms used for analysis. Although *trnH-psbA* consists of a higher number of PIC (Figure S2) than any other gene tested, its power in species discrimination is low (>60%). The two-locus combination of *matK/rbcL* provided an average species resolution of 83%, similar to the three-locus combination of *rbcL/trnH-psbA/matK* with an increased efficiency of 86%. All algorithms showed a good average branch support for the combination of all three loci (Figure 1). As shown in Figure S1, single-locus analysis resulted in different topologies for the obtained trees and resolution as well as support, was low compared to a two-locus combination. The exemplary trees obtained from NJ analysis, indicated the low resolution ability of *rbcL*, while the topology of *matK* is not reflecting the accepted phylogenetic view e.g. for *Syringodium*. The impact of the *matK* sequence data can also be tracked by looking at the two-locus *matK/rbcL* combined tree (Figure 4), especially for *Syringodium* and *Halodule* sp. By taking a detailed look, the *matK/rbcL* tree support for all genera is close to 100%. The more complex genera, *Halodule* and *Halophila* are only partly resolved (*H. beccarii*, *H. stipulacea*) and subpopulations are not distinguishable.

Targeting at a the maximum of informative value in the dataset, it was analyzed for diagnostic characters. Both genes, *matK/rbcL*, were analyzed separately to investigate their usability for character-based barcoding. Furthermore the obtained results were examined for coincidence with the tree-based methods. For *rbcL* the CAOS analysis underlined the results of the tree-based methods (compare Figure S1). For the family and genus level CAs (characteristic attributes) could be found, ranging from 3/599 CAs for *Cymodocea* to 36/599 CAs for *Halodule* (Table S4).

In the *Halophila* complex, only for *H. beccarii* and *H. stipulacea* CAs could be found. The remaining *Halophila* species did not show any difference in their sequence, consequently no CAs were found. The same was true for *Halodule* were no CAs between *H. pinifolia*, *H. uninervis* and *H. wrightii* have been detected. Genetic unique characters were only found for the unknown *Halodule* species in a surprisingly high amount (25/599). In case of the remaining species diagnostic CAs were found as presented in Table S4.

CAs for the families and genera were detected by looking at *matK*,. For most species characteristic attributes could be found which underlines its variability.For the *Halophila* complex, still two blocks remain, bearing the same sequences: (1) *H. ovata*, H. ovalis* and *H.* sp. B, (2) *H. ovalis* and *H. decipiens*. For *H. beccarii* (18/845) sites could be found whereas only (3/845) CAs in *H. stipulacea* were observed. This approach also identified *H.* sp. A as *H. beccarii*. Interestingly, for the subspecies of *H. ovalis* (5/845) CAs were observed. In case of the *Halodule* complex, complete similarity was only detected for *H. uninervis* and *H. pinifolia* from Palk Bay, whereas *Halodule* species from the Chilika lagoon appear to have unique attributes (Table S5).

**Figure 5:**
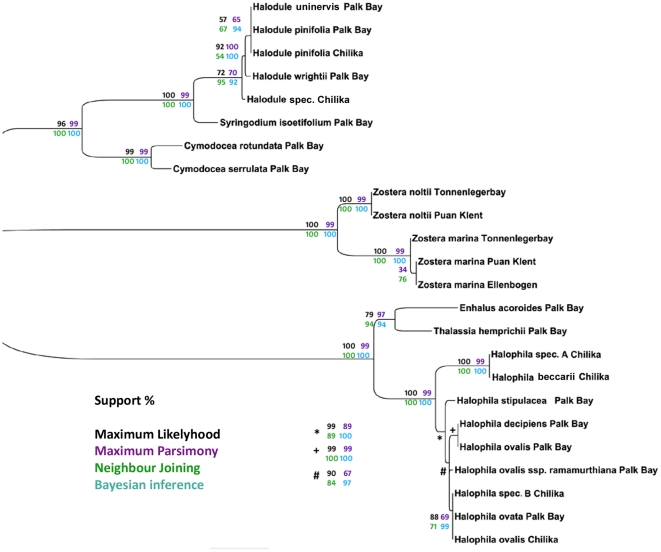
Final consensus tree of all barcode loci and methods. Combined tree of *matK/rbcL/trnH-psbA* loci, branch support values are given in percent. Support values highlighted by the method used: Maximum Likelihood (black), Maximum Parsimony (violet), Neighbor Joining (green) and Bayesian Analysis (turquoise).

The final combined tree resolved three distinct clades of seagrasses: Hydrocharitaceae, Zosteraceae and Cymodoceaceae (Figure 5). The missing family Posidoniaceae is arranged in order to the Cymodoceaceae family in pre-analysis trees (data not shown). The support values of the different nodes are taken from the full-dataset analysis. The results highlight that Bayesian and ML analysis performed mostly similar and less critical than NJ and MP.

The described small genera like *Syringodium*, *Enhalus* and *Thalassia* are well supported and the implied species could be clearly resolved and identified (support ranging from 79 to 100%). The resolution of the complex genera does not improve in comparison to the combination of *rbcL/matK* (Figure 4). A stringent dichotomic tree could not be achieved with the tree-based methods.

For the *Halophila* genus, two unidentified species from the Chilika Lagoon were incorporated into the analysis. The identification based on minor morphological characters is challenging since single leaf-paired *Halophila* species show a high phenotypic plasticity. While *H. beccarii* and *H. stipulacea* form distinct clades, the other *Halophila* species remain as a complex using *matK/rbcL* (Figure 4). Here one unidentified species could be assigned as *H. beccarii* with strong support. Congruent to the two-locus combination, also in the three-locus combination three distinct clades can be found in the inner Halophila complex: (1) *H. ovata* grouped with *H. ovalis* from the Chilika lagoon and the other unknown species; (2) *Halophila ovalis* subsp. *ramamurthiana* and (3) *H. decipiens* and *H. ovalis* from Palk Bay. These three groups are congruent with the findings of the character-based identification approach using *matK* (see Table S5). Consequently, the likelihood of wrong morphological-based assignment can be taken into account.

## Discussion

Recent research indicates seagrasses as a potential alternate source for isolation and extraction of secondary metabolites of high value in medicine and biotechnological applications [58], [59]. Our research aim is to identify and exploit the secondary compounds from seagrasses. For this purpose seagrasses need to be cultured or the valuable compounds need to be synthesized chemically. Based on discussion with Indian scientists, literature studies and on-site visits of Indian seagrass beds, the need for a genetic classification of the investigated species became obvious. At times, separation of different seagrass species becomes challenging, even for a seagrass taxonomist. However, there was the need for a fast, reliable, and cost-efficient system for recognition and identification of seagrasses also by non-experts. In addition there were a number of questions by local ecologists concerning the composition of the seagrass mats including some unassigned species which had not been found before at the specific sites [60]. These kinds of questions can be answered using a technically simple DNA barcoding system.

To establish a DNA barcoding system for seagrasses the recommendations by Hollingsworth et al. [12] for plant species in general were followed using a two-locus approach with specific primers for the amplification of *rbcL* and *matK*. To the best of our knowledge there are no seagrass barcode data available in the CBOL database at present.

Seagrasses expand their meadows by vegetative propagation through rhizomes. Over time individuals can therefore spread over wide areas. Collection of homogeneous seagrass samples is hampered because different genotypes grow in direct vicinity forming vast networks. This was observed for well accessible seagrass locations (e.g. *Z. marina* [31]) populations. Such networks are often multi-specific, comprised of up to 12 species in complex seagrass beds [61] and/or contain different individuals of the same plant species. In addition a voucher specimen should be directly collected and dried to conserve this individual plant also for future references. However, as several people were involved in the collection process, specimen collection of voucher plant individuals could not be ensured. The access to some of the sampling sites was difficult and divers were not always able to follow well-known collection rules. Focus during collection goaled more on finding and identification, than on correct sampling. For the identification of genotypes and their role in the ecosystem the sampling strategy has to be improved and probably one has to concentrate on a small number of species with facile access.

Seagrasses contain a number of different phenolic compounds which make them attractive from a biotechnological point of view, but difficult to handle for genetic analysis from dried material. Several DNA extraction methods have been tested and only a few lead to a DNA quality which could be used for PCR amplifications. Also the PCR conditions have been optimized and several additives were tested to obtain reproducible amplification results. Still the amplification of larger fragments, such as the *matK* fragment of more than 800 bp caused difficulties.

The recommended two-locus DNA barcode consisting of *matK* and *rbcL* [12] was suggested to be the best compromise in comparison to all other loci tested. The results (Figure 4) indicate that two plastid genes are not sufficient to fully resolve seagrasses at the species level. For most projects, a discrimination power of 70% is sufficient, but for higher resolution of complex species, the use of additional loci was supposed [54].

For this project the discrimination power needed to be at a high level so that subspecies level differences can be resolved. This is important for some seagrasses genera where several subspecies and even ecotypes are encountered. Therefore the more variable plastid spacer was included into the analysis. This three-locus combination was successfully used in barcoding tropic plants at diversity hot spots [55], [56]. The *trnH-psbA* spacer, on average about 450 bp, is varying from 296 to 1120 bp based on available data, is one of the most variable plastid regions in angiosperms and is easily amplified across a broad range of land plants [13], [17]. For seagrass species the universality of *matK* and *rbcL* primers is high. After optimization, PCR, cloning and sequencing was straight-forward for all three barcodes used. As expected combined loci analyses dramatically improved the average branch support at different combinations compared to the single-locus analysis. This underlines that single-locus analysis of closely related species does not result in a robust system as already proposed for *matK* [25]. For the two-locus combinations, *rbcL/matK* performed best, while the combinations with *trnH-psbA* where significantly lower in support than in single locus performance. A high value of PICs is not connected with a high discrimination power, at least for sea grass species (Figure 3). The advantage of a three-locus combination is the reproducibility using different algorithms for analysis. Setting the focus on simple and cost effective identification [54] the two-locus combination *matK/rbcL* is adequate as was also demonstrated by the character-based approach. Furthermore the data processing of *trnH-psbA* is not straightforward [52], decreasing the chance for plant barcoding to catch up with fast systems already used for the animal kingdom [62].

Vijayan and Tsou (2010) [13] suggested that until more useful barcodes are identified, the presently proposed DNA barcodes (*rbcL/matK*) can be used to initiate barcoding of all land and water plants, with some family exceptions [18], [21], [22]. This tendency is supported by in our analysis using BLAST as simple and fast identification tool (Table S3). The *matK* and *rbcL* sequences which are already contained in the database provide a good basis for identification at least to the genus level with a database providing comparable data for nearly 70% of the considered seagrass taxa. Since not all species are deposited in publicly available databases, especially questionable subspecies, the combined tree construction is based on the three-locus barcodes. This approach ensures the identification and classification of unknown species (e.g. from the Chilika Lagoon) and *Halophila* subsp. by a trustable average branch support for the different tree constructions methods used. From the consensus tree all genera are separated into clear clades, especially genera with single species (e.g. *Enhalus* or *Thalassia*). For the Zosteraceae, the distance-based methods like MP and NJ could also distinguish subpopulations (e.g. of *Zostera marina*). Still, uncertainties persist with the more complex genera like *Halodule* and *Halophila*. In case of *H. uninervis* the populations of the Chilika lagoon and Palk Bay were distantly out-grouped, while *H. pinifolia* from both areas is grouped together. This is reflecting the confusion and ambiguous status of the taxonomy between these two species [63], although Ito & Tanaka (2011) [64] obtained a clear taxonomy for Asian *Halodule* populations. *H. wrightii* appears to be genetically unique [63], [65], although it is morphologically distinguished only through the form of the leaf tip [1], like *H. pinifolia* and *H. uninervis*.

The character-based barcoding presents a method for unambiguous identification based on analysis of unique sequence sites. This approach is not restricted to DNA-based barcoding applications, nearly every kind of data can be used. For the seagrass dataset no new findings could be elucidated with this approach, but conjectural relations could be verified. The role of *rbcL* as a basic, conservative marker fragment could be underlined as CAs where only found on genus level. The percentage of species identification was similarly low compared to tree-based methods. *MatK* provided much more CAs, archiving the same percentage of species identification as with a tree-based approach. The character-based barcoding indicates additionally the misidentification of *H. decipiens* at Palk Bay and *H. ovalis* at Chilika Lagoon. This misidentification can be considered with high likelihood as it is in congruence with the tree-based approach. Furthermore, the *Halodule* species found at the Lagoon can be verified as genetically different to species found in the open sea. Clear advantage of the character-based system is its correctness, but nevertheless profound bioinformatic knowledge is needed, as the software is based on a compulsory dichotomic tree and a very accurate alignment. Here the identification using BLAST is still the simplest approach.

It is important to understand genetic differences and population structure to sustain current level genetic diversity within the *Halodule* species. Based on this point the plants collected at Chilika Lagoon could represent a species or subspecies of *Halodule* as it was out-grouped separately from all three *Halodule* species of India. One cannot refuse the probable genetic mix-up among the species. Our data indicate this possibility especially in case of *H. pinifolia* and *H. uninervis*. Furthermore, the hit number for *rbcL* was ambiguous for *H. uninervis* as well as *H. pinifolia* while performing the BLAST identification approach. This indicates that more detailed genetic studies among the different populations of *Halodule* in India are needed, similar to the approach of Ito & Tanaka (2011) [64] for Asian *Halodule* populations.

In case of the *Halophila* genus the position of *H. decipiens* is not correct. In previous studies *H. decipiens* was found on the base of the *Halophila* clade [32], [34], indicating a potential genetic mix-up between indo-pacific species, possibly by cross breeding. This is supported by its worldwide distribution, where no sequence differences occur between samples from the Caribbean and from around the Indo-Pacific [63]. *H. ovalis* and *H. ovata* could be differentiated only with leaf lamina and number of leaf veins, but confusions persist in identification of juveniles. Comparison to previous sequencing results could be only tested using *rbcL*, showing a high overlap (Table S3).

In case of the simple thallus-like *Halophila* species, the *rbcL* sequence characters are as similar as their morphological characters and do not provide an informative content for barcoding (see Table S4 and S5). In particular, misidentifications in the *Halophila* genus could be elucidated with the presented barcoding approach, though this could not be achieved for *Halodule*. DNA barcoding is wrongly addressed to this taxonomically problematic genus. The presented barcoding system verifies that these two genera are challenging for identification. So for establishing a correct seagrass barcoding database, close ups like microsatellites should be used, as recently published for *H. beccarii* [66] rather than misleading, environmentally influenced morphological characters.

This could uncover whether a genetic mix-up occurred or phenotypic plasticity is an underestimated trait for seagrasses [8], helping them to survive in different niches like deep water, deepwater with light limitation, intertidal areas, shallow turbid waters, eutrophic waters etc. where they are capable of adapting to overcome the local conditions. Only more detailed loci may solve the complex taxonomy and cleanup questionable assignments for species which share nearly all morphological identities like *H. ovata*, *H. gaudichaudii* and *H. okinawensis* [34]. To summarize the results of the different trees, it is obvious that a single locus is not able to resolve well-described species properly (*rbcL*, *trnH-psbA*) or the marker is not robust using only a few specimens (*matK*). Hence, one gets a result not fitting to the well-known phylogeny of Alismatales.

A rapid system for identification close to species level is provided by using *matK* and *rbcL*. The spacer region *trnH-psbA* cannot be recommended for barcoding purposes at least for the ecological group of seagrasses. The fact of a clearly existing “barcoding gap” makes it not suitable for barcoding seagrass, not providing more information than e.g. *rbcL* or *matK*. Including the structure of internal spacers could be a promising approach for phylogenetic analysis. ITS or *phyC* could serve as additional supplemental loci and were used successfully in several other phylogenetic analyses [33], [34], [63]. One should be aware that barcoding is not a replacement for phylogenetic analysis and maybe the remaining complexes of *Halodule* and *Halophila* are a more taxonomic and systematic problem and it is wrongly addressed by a simple method like DNA barcoding. The complex formations of *Halodule* and *Halophila* need to be more carefully analyzed as they show more phenotypic plasticity and are known to be problematic concerning species discrimination [6], [34], [64].

In summary, recognition of seagrasses by DNA barcoding is possible and feasible. Two unknown species from the Chilika Lagoon could successfully be identified (*H. beccarii and H. ovata*) and were verified on the congruence of tree- and character-based analysis. Misidentifications can be unambiguously defined as the bottleneck for establishing a solid database for DNA-based identification of seagrass. Furthermore these misidentifications, e.g. *H. ovalis* from the Chilika Lagoon or *H. decipiens* from Palk Bay, could be revealed by the developed system. The known complex of *Halodule* remained, unfortunately, unresolved. This indicates, as discussed above, the demand for a more detailed study including worldwide populations and the choice of a high resolution marker system (e.g. microsatellites) [66]. Our recommendation is to use the *matK/rbcL* combination as a cost effective and straight forward method. The attained identification level is good enough for ecological survey and conservation purposes, as meadows constantly decrease in area and species diversity.

## Supporting Information

Figure S1 **Comparison of** ***rbcL*** **and** ***matK*** **tree using Neighbor Joining (NJ) method.** Trees resulting from NJ analysis (1,000 Bootstrap replicates) of *rbcL* and *matK* dataset compared using TreeJuxtaposer [48]. Red lines and taxons in red indicate different topology, letters in green topology in the compared tree. Collection sites other than Palk Bay are marked as follows: *Tonnenlegerbay, **Puan Klent, ***Ellenbogen and ° Chilika Lagoon. *H. ovalis* subsp. *ramamurthiana* is abbreviated as *H. ovalis* subsp.
(TIF)

Figure S2 **Dataset composition.** The composition of the single- and combined-loci was analyzed for the percentage of conserved, variable, singleton and parsimony informative characters (PIC). *rbcL* shows the highest content of conserved sites, *psbA* spacer the lowest and opposite in the case of diverged characters. Combination of loci revealed in an average content of ∼24% PIC, *psbA+matK* with 28.4% PIC, and *matK+rbcL* and *rbcL+psbA* 22% PIC.
(TIF)

Figure S3 **Inverted repeats (IR) in the** ***trnH-psbA*** **spacer and its conservation in seagrasses.** Parts incorporating the recognized IR sequence from the *trnH-psbA* alignment, IRs are shaded in grey color. A) Conserved IR in *Zostera* and *Syringodium*, close to the 3′ prime end. B) Conserved IR in *Halodule* and *Halophila*, showing a non-conserved part in *Zostera* but nearly full conservation in *Syringodium*.
(TIF)

Table S1 **Deposited accession numbers and collection places with GPS data for all observed specimens, as well as downloaded accession numbers for comparison (see Table S3).**
(DOCX)

Table S2 **Primer universality for each locus used.** Performance of all six primer combinations used on the 24 species in the dataset and five additional herbarium species.
(DOCX)

Table S3 **Identification level using BLAST and overlap percentage with the already deposited sequences on NCBI database.** Sequences of species already available on NCBI are labeled in grey color, for those overlap was estimated in percent. Identification level is coded as following: O order, F family, G genus and S species level. Origins indicated as follows * Chilika, ** Ellenbogen, *** Puan Klent.
(DOCX)

Table S4 **Diagnosis of Characteristic Attributes (CAs) for the** ***rbcL*** **fragment.** Diagnostic characters for each genus (number of included species) or species are listed with position and respective nucleotide. SNP analysis was carried out using CAOS software.
(DOCX)

Table S5 **Diagnosis of Characteristic Attributes (CAs) for the** ***matK*** **fragment.** Diagnostic characters for the identified species are listed with position and respective nucleotide. SNP analysis was carried out using CAOS software. Species marked with an asterisk (*) originate from the Chilika Lagoon. Sequences with complete similarity are marked as included (incl.).
(DOCX)

## References

[1] den Hartog C (1970).

[2] Bremer K, Nordenstam B, ElGhazaly G, Kassas M (2000). Phylogenetic nomenclature and the new ordinal system of the angiosperms.. Plant Systematics for the 21st Century.

[3] Janssen T, Bremer K (2004). The age of major monocot groups inferred from 800+rbcL sequences.. Botanical Journal of the Linnean Society.

[4] Kato Y, Aioi K, Omori Y, Takahata N, Satta Y (2003). Phylogenetic analyses of *Zostera* species based on *rbcL* and *matK* nucleotide sequences: Implications for the origin and diversification of seagrasses in Japanese waters.. Genes & Genetic Systems.

[5] Les DH, Cleland MA, Waycott M (1997). Phylogenetic studies in Alismatidae, II: Evolution of marine angiosperms (seagrasses) and hydrophily.. Systematic Botany.

[6] Waycott M, Duarte CM, Carruthers TJB, Orth RJ, Dennison WC (2009). Accelerating loss of seagrasses across the globe threatens coastal ecosystems.. Proceedings of the National Academy of Sciences of the United States of America.

[7] Reusch TBH, Hukriede W, Stam WT, Olsen JL (1999). Differentiating between clonal growth and limited gene flow using spatial autocorrelation of microsatellites.. Heredity.

[8] Bricker E, Waycott M, Calladine A, Zieman JC (2011). High connectivity across environmental gradients and implications for phenotypic plasticity in a marine plant.. Marine Ecology-Progress Series.

[9] den Hartog C, Kuo J (2006). Taxonomy and biogeography of seagrasses *in* Seagrass Biology, Springer, New York,.

[10] von Cräutlein M, Korpelainen H, Pietiläinen M, Rikkinen J (2011). DNA barcoding: a tool for improved taxon identification and detection of species diversity.. Biodiversity and Conservation.

[11] Kress WJ, Wurdack KJ, Zimmer EA, Weigt LA, Janzen DH (2005). Use of DNA barcodes to identify flowering plants.. Proceedings of the National Academy of Sciences of the United States of America.

[12] Hollingsworth PM, Forrest LL, Spouge JL, Hajibabaei M, Ratnasingham S (2009b). A DNA barcode for land plants.. Proceedings of the National Academy of Sciences of the United States of America.

[13] Vijayan K, Tsou CH (2010). DNA barcoding in plants: taxonomy in a new perspective.. Current Science.

[14] Hebert PDN, Cywinska A, Ball SL, DeWaard JR (2003). Biological identifications through DNA barcodes.. Proceedings of the Royal Society of London Series B-Biological Sciences.

[15] Chase MW, Cowan RS, Hollingsworth PM, van den Berg C, Madrinan S (2007). A proposal for a standardised protocol to barcode all land plants.. Taxon.

[16] Fazekas AJ, Burgess KS, Kesanakurti PR, Graham SW, Newmaster SG (2008). Multiple multilocus DNA barcodes from the plastid genome discriminate plant species equally well.. Plos One.

[17] Kress WJ, Erickson DL (2007). A Two-Locus Global DNA Barcode for Land Plants: The coding *rbcL* gene complements the non-coding *trnH-psbA* spacer region.. Plos One.

[18] Roy S, Tyagi A, Shukla V, Kumar A, Singh UM (2010). Universal plant DNA barcode loci may not work in complex groups: A Case Study with Indian *Berberis* Species.. Plos One.

[19] Shaw J, Lickey EB, Beck JT, Farmer SB, Liu WS (2005). The tortoise and the hare II: Relative utility of 21 noncoding chloroplast DNA sequences for phylogenetic analysis.. American Journal of Botany.

[20] Shaw J, Lickey EB, Schilling EE, Small RL (2007). Comparison of whole chloroplast genome sequences to choose noncoding regions for phylogenetic studies in angiosperms: The tortoise and the hare III.. American Journal of Botany.

[21] Seberg O, Petersen G (2009). How many loci does it take to DNA barcode a Crocus?. Plos One.

[22] Spooner DM (2009). DNA barcoding will frequently fail in complicated groups: an example in wild potatoes.. American Journal of Botany.

[23] Wang WQ, Wu YR, Yan YH, Ermakova M, Kerstetter R (2010). DNA barcoding of the Lemnaceae, a family of aquatic monocots.. Bmc Plant Biology.

[24] Pillon Y, Munzinger J, Amir H, Hopkins HCF, Chase MW (2009). Reticulate evolution on a mosaic of soils: diversification of the New Caledonian endemic genus *Codia* (Cunoniaceae).. Molecular Ecology.

[25] Lahaye R, Van der Bank M, Bogarin D, Warner J, Pupulin F (2008). DNA barcoding the floras of biodiversity hotspots.. Proceedings of the National Academy of Sciences of the United States of America.

[26] Kress WJ, Erickson DL (2008). DNA barcodes: Genes, genomics, and bioinformatics.. Proceedings of the National Academy of Sciences of the United States of America.

[27] Goldstein PZ, DeSalle R (2011). Integrating DNA barcode data and taxonomic practice: Determination, discovery, and description.. Bioessays.

[28] DeSalle R, Egan MG, Siddall M (2005). The unholy trinity: taxonomy, species delimitation and DNA barcoding.. Philosophical Transactions of the Royal Society B-Biological Sciences.

[29] Barrett SCH, Eckert CG, Husband BC (1993). Evolutionary processes in aquatic plant-populations.. Aquatic Botany.

[30] Tanaka N, Setoguchi H, Murata J (1997). Phylogeny of the family Hydrocharitaceae inferred from *rbcL* and *matK* gene sequence data.. Journal of Plant Research.

[31] Procaccini G, Olsen JL, Reusch TBH (2007). Contribution of genetics and genomics to seagrass biology and conservation.. Journal of Experimental Marine Biology and Ecology.

[32] Waycott M, Freshwater DW, York RA, Calladine A, Kenworthy WJ (2002). Evolutionary trends in the seagrass genus *Halophila* (thouars): Insights from molecular phylogeny.. Bulletin of Marine Science.

[33] Les DH, Moody ML, Jacobs SWL, Bayer RJ (2002). Systematics of seagrasses (Zosteraceae) in Australia and New Zealand.. Systematic Botany.

[34] Uchimura M, Faye EJ, Shimada S, Inoue T, Nakamura Y (2008). A reassessment of Halophila species (Hydrocharitaceae) diversity with special reference to Japanese representatives.. Botanica Marina.

[35] Short FT, Moore GE, Peyton KA (2010). *Halophila ovalis* in the Tropical Atlantic Ocean.. Aquatic Botany.

[36] Spielman D, Brook BW, Frankham R (2004). Most species are not driven to extinction before genetic factors impact them.. Proceedings in National Academic Science U S A.

[37] Jones TC, Gemmill CEC, Pilditch CA (2008). Genetic variability of New Zealand seagrass (Zostera muelleri) assessed at multiple spatial scales.. Aquatic Botany.

[38] Storchova H, Hrdlickova R, Chrtek J, Tetera M, Fitze D (2000). An improved method of DNA isolation from plants collected in the field and conserved in saturated NaCl/CTAB solution.. Taxon.

[39] Ford CS, Ayres KL, Toomey N, Haider N, Stahl JV (2009). Selection of candidate coding DNA barcoding regions for use on land plants.. Botanical Journal of the Linnean Society.

[40] Stanford AM, Harden R, Parks CR (2000). Phylogeny and biogeography of *Juglans* (Juglandaceae) based on *matK* and ITS sequence data.. American Journal of Botany.

[41] White TJ, Bruns T, Lee S, Taylor JW (1990).

[42] Sang T, Crawford DJ, Stuessy TF (1997). Chloroplast DNA phylogeny, reticulate evolution, and biogeography of *Paeonia* (Paeoniaceae).. American Journal of Botany.

[43] Tate JA, Simpson BB (2003). Paraphyly of *Tarasa* (Malvaceae) and diverse origins of the polyploid species.. Systematic Botany.

[44] Hall TA (1999). BioEdit: a user-friendly biological sequence alignment editor and analysis program for Windows 95/98/NT.. Nucleic Acids Symposium Series.

[45] Posada D (2008). jModelTest: Phylogenetic model averaging.. Molecular Biology and Evolution.

[46] Ronquist F, Huelsenbeck JP (2003). MrBayes 3: Bayesian phylogenetic inference under mixed models.. Bioinformatics.

[47] Bergmann T, Hadrys H, Breves G, Schierwater B (2009). Character-based DNA barcoding: a superior tool for species classification.. Berliner und Münchener Tierarztliche Wochenschrift.

[48] Tamura K, Peterson D, Peterson N, Stecher G, Nei M, Kumar S (2011). MEGA5: Molecular evolutionary genetics analysis using Maximum Likelihood, Evolutionary Distance, and Maximum Parsimony methods.. Molecular Biology and Evolution.

[49] Meier R, Shiyang K, Vaidya G, Ng PKL (2006). DNA barcoding and taxonomy in diptera: A tale of high intraspecific variability and low identification success.. Systematic Biology.

[50] Guindon S, Gascuel O (2003). A simple, fast, and accurate algorithm to estimate large phylogenies by maximum likelihood.. Systematic Biology.

[51] Munzner T, Guimbretiere F, Tasiran S, Zhang L, Zhou YH (2003). TreeJuxtaposer: Scalable tree comparison using Focus+Context with guaranteed visibility.. Acm Transactions on Graphics.

[52] Whitlock BA, Hale AM, Groff PA (2010). Intraspecific inversions pose a challenge for the *trnH-psbA* plant DNA barcode.. Plos One.

[53] Rice P, Longden I, Bleasby A (2000). EMBOSS: The European molecular biology open software suite.. Trends in Genetics.

[54] Hollingsworth ML, Clark AA, Forrest LL, Richardson J, Pennington RT (2009a). Selecting barcoding loci for plants: evaluation of seven candidate loci with species-level sampling in three divergent groups of land plants.. Molecular Ecology Resources.

[55] Kress WJ, Erickson DL, Jones FA, Swenson NG, Perez R (2009). Plant DNA barcodes and a community phylogeny of a tropical forest dynamics plot in Panama.. Proceedings of the National Academy of Sciences of the United States of America.

[56] Schreeg LA, Kress WJ, Erickson DL, Swenson NG (2010). Phylogenetic Analysis of Local-Scale Tree Soil Associations in a Lowland Moist Tropical Forest.. Plos One.

[57] Meyer CP, Paulay G (2005). DNA barcoding: Error rates based on comprehensive sampling.. Plos Biology.

[58] Newby BMZ, Cutright T, Barrios CA, Xu QW (2006). Zosteric acid - An effective antifoulant for reducing fresh water bacterial attachment on coatings.. Journal of coatings technology and research.

[59] Achamlale S, Rezzonico B, Grignon-Dubois M (2009). Evaluation of Zostera detritus as a potential new source of zosteric acid.. Journal of Applied Phycology.

[60] Ghosh A, Pattnaik AK, Ballatore TJ (2006). Chilika Lagoon: Restoring ecological balance and livelihoods through re-salinization.. Lakes & Reservoirs: Research & Management.

[61] Duarte CM (2000). Marine biodiversity and ecosystem services: an elusive link.. Journal of Experimental Marine Biology and Ecology.

[62] Ivanova NV, Borisenko AV, Hebert PDN (2009). Express barcodes: racing from specimen to identification.. Molecular Ecology Resources.

[63] Waycott M, Procaccini G, Les DH, Reusch T (2006). A genetic perspective in seagrass evolution, ecology and conservation.. Springer Academic, Netherlands.

[64] Ito Y, Tanaka N (2011). Hybridisation in a tropical seagrass genus, *Halodule* (Cymodoceaceae), inferred from plastid and nuclear DNA phylogenies.. Telopea.

[65] Angel R (2002). Genetic diversity of *Halodule wrightii* using random amplified polymorphic DNA.. Aquatic Botany.

[66] Jiang K, Shi YS, Zhang J, Xu NN (2011). Microsatellite primers for vulnerable seagrass *Halophila beccarii* (Hydrocharitaceae).. American Journal of Botany.

