# Supplementary figures and images for: Development of a DNA Barcoding System for Seagrasses: Successful but Not Simple
Source: PLoS One. 2012 Jan 11;7(1):e29987. doi: 10.1371/journal.pone.0029987 (PMC3256190; doi:10.1371/journal.pone.0029987)

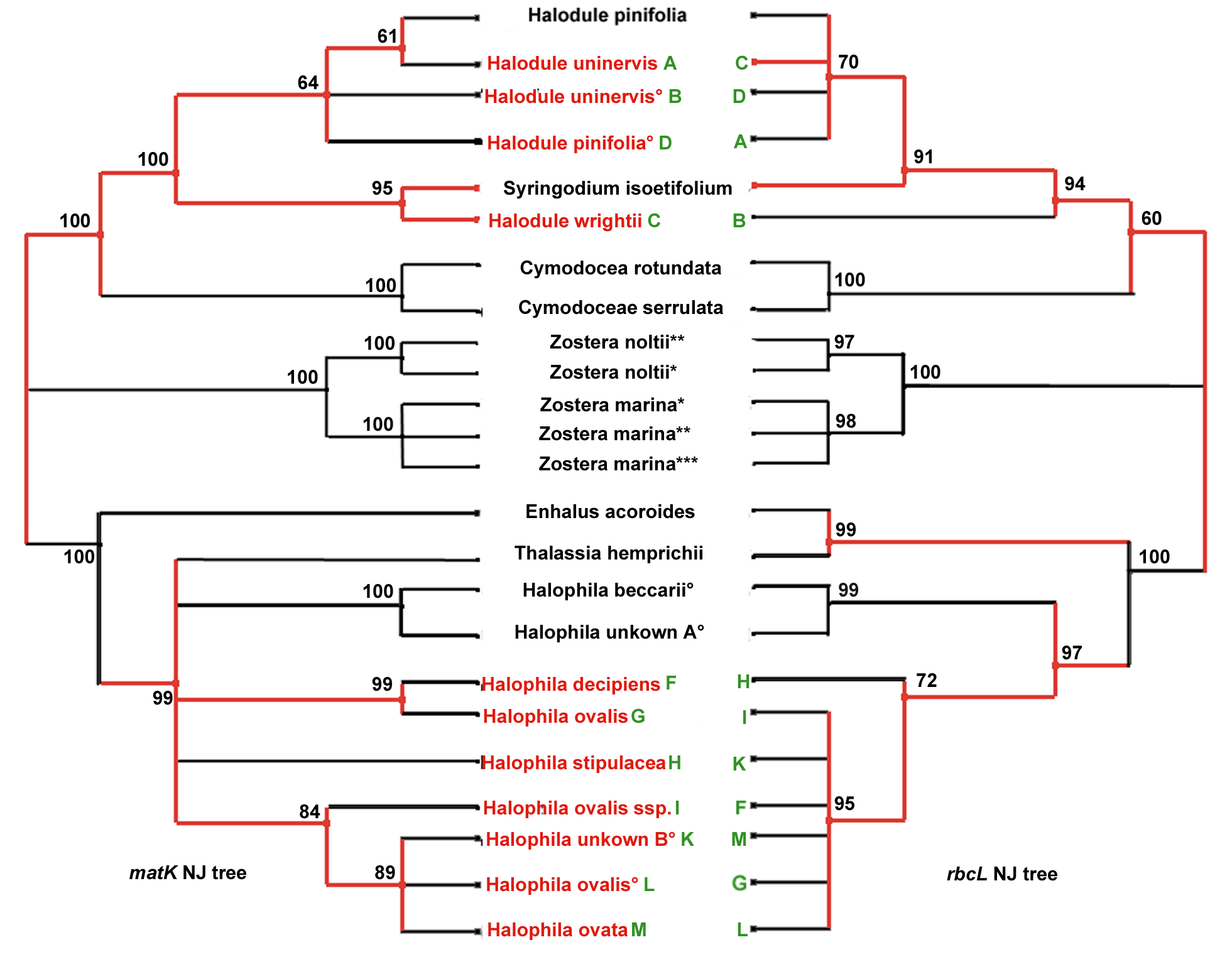

Supplement: Figure S1 — Comparison of rbcL and matK tree using Neighbor Joining (NJ) method. Trees resulting from NJ analysis (1,000 Bootstrap replicates) of rbcL and matK dataset compared using TreeJuxtaposer [48]. Red lines and taxons in red indicate different topology, letters in green topology in the compared tree. Collection sites other than Palk Bay are marked as follows: *Tonnenlegerbay, **Puan Klent, ***Ellenbogen and ° Chilika Lagoon. H. ovalis subsp. ramamurthiana is abbreviated as H. ovalis subsp. (TIF) [file pone.0029987.s001.tif]

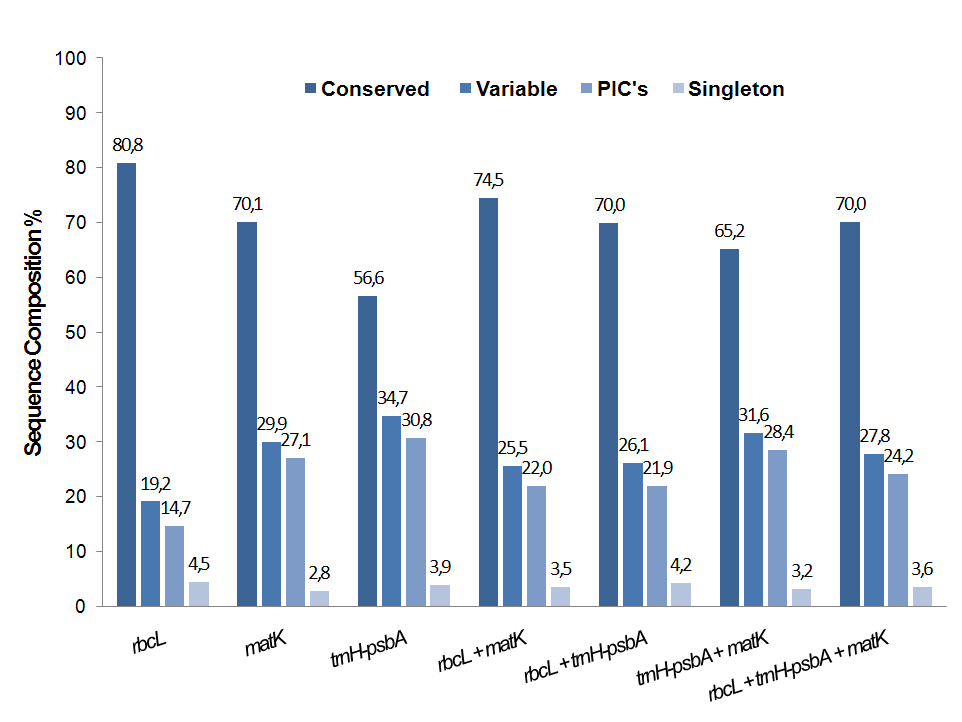

Supplement: Figure S2 — Dataset composition. The composition of the single- and combined-loci was analyzed for the percentage of conserved, variable, singleton and parsimony informative characters (PIC). rbcL shows the highest content of conserved sites, psbA spacer the lowest and opposite in the case of diverged characters. Combination of loci revealed in an average content of ∼24% PIC, psbA+matK with 28.4% PIC, and matK+rbcL and rbcL+psbA 22% PIC. (TIF) [file pone.0029987.s002.tif]

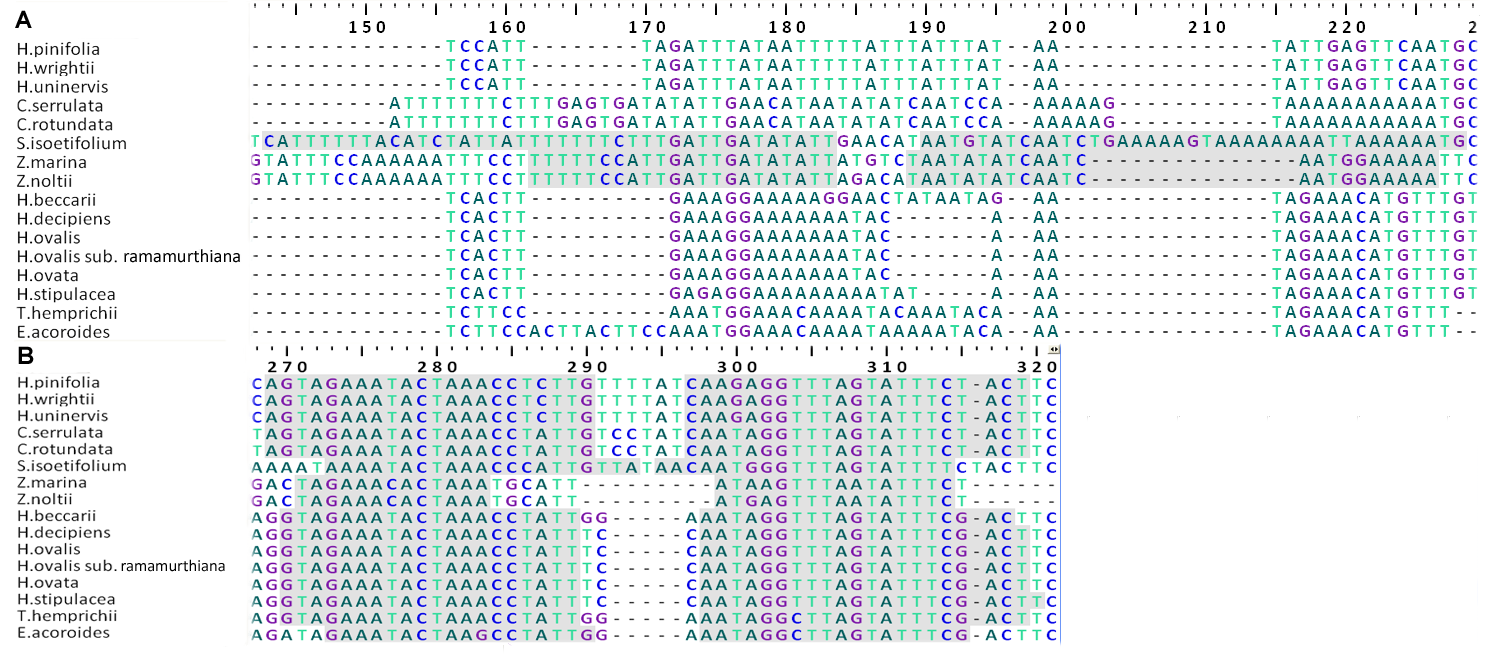

Supplement: Figure S3 — Inverted repeats (IR) in the trnH-psbA spacer and its conservation in seagrasses. Parts incorporating the recognized IR sequence from the trnH-psbA alignment, IRs are shaded in grey color. A) Conserved IR in Zostera and Syringodium, close to the 3′ prime end. B) Conserved IR in Halodule and Halophila, showing a non-conserved part in Zostera but nearly full conservation in Syringodium. (TIF) [file pone.0029987.s003.tif]
