# Supplementary material for: Development of a DNA Barcoding System for Seagrasses: Successful but Not Simple
Source: PLoS One. 2012 Jan 11;7(1):e29987. doi: 10.1371/journal.pone.0029987 (PMC3256190; doi:10.1371/journal.pone.0029987)
Supplement: Table S1 — Deposited accession numbers and collection places with GPS data for all observed specimens, as well as downloaded accession numbers for comparison (see Table S3). (DOCX) [file pone.0029987.s004.docx]

|  | | | | | | | | | |
| --- | --- | --- | --- | --- | --- | --- | --- | --- | --- |
|  |  |  |  | ***Accession numbers*** | | | ***Accession numbers used for comparison*** | | |
| ***Species*** | ***Place of sampling*** | ***GPS coordinates*** | ***Collector*** | ***rbcL*** | ***matK*** | ***trnH-psbA*** | ***rbcL*** | ***matK*** | ***trnH-psbA*** |
| *H. ovalis* | *Chilika Lagoon, India* | *LAT:19,66 LON:85,20* | *J. Papenbrock* | *JN225349* | *JN225365* | *JN225325* | *AB004890* | *AB002570.1* | [*GU906229.1*](http://www.ncbi.nlm.nih.gov/nucleotide/292398057?report=genbank&log$=nucltop&blast_rank=1&RID=Y9DVZ1XE01S) |
|  | *Palk Bay, India* | *LAT: 8,61 LON:79,89* | *T. Thangaradjou* | *JN225348* | *JN225366* | *JN225316* | *AB004890* | *AB002570.1* | [*GU906229.1*](http://www.ncbi.nlm.nih.gov/nucleotide/292398057?report=genbank&log$=nucltop&blast_rank=1&RID=Y9DVZ1XE01S) |
| subsp*. ramamurthiana.* | *Palk Bay, India* | *LAT: 9,31 LON:78,10* | *T. Thangaradjou* | *JN225355* | *JN225380* | *JN225332* |  |  |  |
| *H. ovata* | *Palk Bay, India* | *LAT:10,71 LON:70,10* | *T. Thangaradjou* | *JN225347* | *JN225367* | *JN225315* |  |  |  |
| *H. decipiens* | *Palk Bay, India* | *LAT:11,62 LON:69,70* | *T. Thangaradjou* | *JN225340* | *JN225364* | *JN225318* | *HDU80698* |  |  |
| *H. becarrii* | *Chilika Lagoon, India* | *LAT:19,66 LON:85,20* | *J. Papenbrock* | *JN225339* | *JN225363* | *JN225321* |  |  |  |
| *H. stipulacea* | *Palk Bay, India* | *LAT: 9,38 LON:77,10* | *T. Thangaradjou* | *JN225356* | *JN225381* | *JN225333* |  |  |  |
| *H.* spec. *A* | *Chilika Lagoon, India* | *LAT:19,66 LON:85,20* | *J. Papenbrock* | *JN225337* | *JN225361* | *JN225323* |  |  |  |
| *H.* spec*. B* | *Chilika Lagoon, India* | *LAT:19,66 LON:85,20* | *J. Papenbrock* | *JN225338* | *JN225362* | *JN225324* |  |  |  |
| *T. hemprichii* | *Palk Bay, India* | *LAT:10,71 LON:79,10* | *T. Thangaradjou* | *JN225341* | *JN225373* | *JN225313* | *U80710.1* | *AB002577* |  |
| *E. acoroides* | *Palk Bay, India* | *LAT: 9,38 LON:78,10* | *T. Thangaradjou* | *JN225336* | *JN225360* | *JN225312* | *U80697* | *AB002569.1* |  |
| *C. rotundata* | *Palk Bay, India* | *LAT:10,71 LON:79,10* | *T. Thangaradjou* | *JN225334* | *JN225358* | *JN225310* |  |  |  |
| *C. serrulata* | *Palk Bay, India* | *LAT:10,71 LON:79,10* | *T. Thangaradjou* | *JN225335* | *JN225359* | *JN225311* | *U80687.1* |  | *FJ648790.1* |
| *H. pinifolia* | *Chilika Lagoon, India* | *LAT:19,66 LON:85,20* | *J. Papenbrock* | *JN225345* | *JN225369* | *JN225322* | *AB571205* |  | *AB571189.1* |
|  | *Palk Bay, India* | *LAT:10,71 LON:79,10* | *T. Thangaradjou* | *JN225346* | *JN225368* | *JN225317* | *AB571205* |  | *AB571189.1* |
| *H.* spec*.* | *Chilika Lagoon, India* | *LAT:19,66 LON:85,20* | *J. Papenbrock* | *JN225343* | *JN225371* | *JN225320* | [*AB571206.1*](http://www.ncbi.nlm.nih.gov/nucleotide/300392653?report=genbank&log$=nucltop&blast_rank=6&RID=Y7MPX2D801N) | *AY952424.1* | *AB571191.1* |
| *H. uninervis* | *Palk Bay, India* | *LAT:10,71 LON:79,10* | *T. Thangaradjou* | *JN225344* | *JN225370* | *JN225319* | [*AB571206.1*](http://www.ncbi.nlm.nih.gov/nucleotide/300392653?report=genbank&log$=nucltop&blast_rank=6&RID=Y7MPX2D801N) | *AY952424.1* | *AB571191.1* |
| *H. wrightii* | *Palk Bay, India* | *LAT:10,71 LON:79,10* | *T. Thangaradjou* | *JN225357* | *JN225379* | *JN225331* | *AB571197* |  |  |
| *S. isoetifolium* | *Palk Bay, India* | *LAT: 9,38 LON:78,10* | *T. Thangaradjou* | *JN225342* | *JN225372* | *JN225314* | *AB507901.1* |  | *FJ648793.1* |
| *Z. marina* | *Sylt, Germany* | *LAT: 55,05 LON:8,45* | *J. Papenbrock* | *JN225354* | *JN225374* | *JN225326* | *AB125349* | *AB125356.1* | *DQ786516.1* |
|  | *Sylt, Germany* | *LAT: 55,03 LON:8,40* | *J. Papenbrock* | *JN225352* | *JN225378* | *JN225328* | *AB125349* | *AB125356.1* | *DQ786516.1* |
|  | *Sylt, Germany* | *LAT: 54,97 LON:8,35* | *J. Papenbrock* | *JN225353* | *JN225375* | *JN225327* | *AB125349* | *AB125356.1* | *DQ786516.1* |
| *Z. noltii* | *Sylt, Germany* | *LAT: 55,03 LON:8,40* | *J. Papenbrock* | *JN225350* | *JN225377* | *JN225330* | *U80733* | *AB096170.1* |  |
|  | *Sylt, Germany* | *LAT: 54,97 LON:8,35* | *J. Papenbrock* | *JN225351* | *JN225376* | *JN225329* | *U80733* | *AB096170.1* |  |
