# Supplementary material for: Development of a DNA Barcoding System for Seagrasses: Successful but Not Simple
Source: PLoS One. 2012 Jan 11;7(1):e29987. doi: 10.1371/journal.pone.0029987 (PMC3256190; doi:10.1371/journal.pone.0029987)
Supplement: Table S2 — Primer universality for each locus used. Performance of all six primer combinations used on the 24 species in the dataset and five additional herbarium species. (DOCX) [file pone.0029987.s005.docx]

| **Primer pair** | **Gene fragment**  **(source)** | **PCR success** | **Species** | **Obtained fragment length** |
| --- | --- | --- | --- | --- |
| P609/P610 | *rbcL* (cp) | 86.21 % | 25 of 29 | 599 bp |
| P608/P607 | *matK* (cp) | 48.28 % | 14 of 29 | 889 bp |
| P646/P647 | *matK* (cp) | 82.76 % | 24 of 29 | 945 bp |
| P672/P673 | *trnQ-rps16* spacer (cp) | 86.21 % | 25 of 29 | ~900 - 1600 bp |
| P676/P677 | *trnH-psbA* spacer (cp) | 93.10 % | 27 of 29 | ~230 – 450 bp |
| P674/P675 | ITS spacer (nuc) | 82.76 % | 24 of 29 | ~800 - 1400 bp |
