# Supplementary material for: Development of a DNA Barcoding System for Seagrasses: Successful but Not Simple
Source: PLoS One. 2012 Jan 11;7(1):e29987. doi: 10.1371/journal.pone.0029987 (PMC3256190; doi:10.1371/journal.pone.0029987)
Supplement: Table S3 — Identification level using BLAST and overlap percentage with the already deposited sequences on NCBI database. Sequences of species already available on NCBI are labeled in grey color, for those overlap was estimated in percent. Identification level is coded as following: O order, F family, G genus and S species level. Origins indicated as follows * Chilika, ** Ellenbogen, *** Puan Klent. (DOCX) [file pone.0029987.s006.docx]

|  | **ID Level** | | | **Overlap %** | | |
| --- | --- | --- | --- | --- | --- | --- |
|  | ***matK*** | ***rbcL*** | ***trnH-psbA*** | ***matK*** | ***rbcL*** | ***trnH-psbA*** |
| *C. rotundata* | O | G | O |  |  |  |
| *C. serrulata* | O | S | O |  | 100.0 | 88.8 |
| *E. acoroides* | S | S | F | 99.6 | 99.8 |  |
| *H. beccarii** | G | G | G |  |  |  |
| *H. decipiens* | G | S | G |  | 99.3 |  |
| *H. ovalis* | S | G | S | 99.1 | 99.7 | 98.1 |
| *H. ovalis* subsp. | G | G | G |  |  |  |
| *H. ovalis** | S | G | S | 98.6 | 99.7 | 98.1 |
| *H. ovata* | G | G | G |  |  |  |
| *H. pinifolia* | O | S | S |  | 100.0 | 99.7 |
| *H. pinifolia** | O | S | S |  | 99.8 | 99.7 |
| *H. stipulacea* | G | G | G |  |  |  |
| *H. uninervis* | O | G | G | 86.3 | 98.6 | 98.2 |
| *H.* spec. *** | O | G | G | 85.9 | 94.8 | 98.5 |
| *H.* spec. A* | G | G | G |  |  |  |
| *H*. spec. B* | G | G | G |  |  |  |
| *H. wrightii* | O | G | G |  | 99.1 |  |
| *S. isoetifolium* | O | F | S |  | 97.9 | 98,41 |
| *T. hemprichii* | S | G | F | 99.2 |  |  |
| *Z. marina* | S | G | S | 100.0 | 100.0 | 99.8 |
| *Z. marina*** | S | G | S | 100.0 | 99.8 | 99.8 |
| *Z. marina**** | S | G | S | 100.0 | 99.8 | 99.8 |
| *Z. noltii* | S | G | G | 99.2 | 99.8 |  |
| *Z. noltii**** | S | G | G | 99.2 | 99.8 |  |
