# Supplementary material for: Development of a DNA Barcoding System for Seagrasses: Successful but Not Simple
Source: PLoS One. 2012 Jan 11;7(1):e29987. doi: 10.1371/journal.pone.0029987 (PMC3256190; doi:10.1371/journal.pone.0029987)
Supplement: Table S4 — Diagnosis of Characteristic Attributes (CAs) for the rbcL fragment. Diagnostic characters for each genus (number of included species) or species are listed with position and respective nucleotide. SNP analysis was carried out using CAOS software. (DOCX) [file pone.0029987.s007.docx]

| **Genus** | **Characteristic attributes** |
| --- | --- |
| *Cymodocea* (2) | 135(T), 328(A), 459(C) |
| *Halophila* (6) | 173(C), 177(A), 234(C), 282(A), 315(C), 399(G), 441(C), 471(A), 473 (A), 474(A) |
| *Halodule* (3) | 21(C), 90(C), 92(A), 96(G), 99(A), 102(A), 105(A), 108(C), 118(T), 126(C), 129(A), 132(C), 147(C), 188(G), 198(A), 210(T), 231(G), 258(G), 294(G), 324(C), 336(C), 366(A), 369(C), 371(A), 393(T), 414(A), 435(G), 453(T), 468(A), 492(G), 519(A), 540(G), 549(A), 560(T), 561(G), 573(T) |
| *Zostera* (2) | 144(G), 192 197(G), 208(G), 209(A), 228(C), 267(C), 297(G), 299(T), 310(A), 317 321(A), 322(G), 323(T), 354(C), 417(G), 462(C), 504(C), 547(C), 568(G), 572(T) |
| *Thalassia* (1) | 45(A), 176(G), 353(T), 372(G), 438(C) |
| *Enhalus* (1) | 45(G), 176(A), 353(G), 372(A), 438(A) |
| *Syringodium* (1) | 72(T), 133 (G) |
