# Supplementary material for: Development of a DNA Barcoding System for Seagrasses: Successful but Not Simple
Source: PLoS One. 2012 Jan 11;7(1):e29987. doi: 10.1371/journal.pone.0029987 (PMC3256190; doi:10.1371/journal.pone.0029987)
Supplement: Table S5 — Diagnosis of Characteristic Attributes (CAs) for the matK fragment. Diagnostic characters for the identified species are listed with position and respective nucleotide. SNP analysis was carried out using CAOS software. Species marked with an asterisk (*) originate from the Chilika Lagoon. Sequences with complete similarity are marked as included (incl.). (DOCX) [file pone.0029987.s008.docx]

| **Species** | **Characteristic attributes** |
| --- | --- |
| *C. serrulata* | 48(A), 105(T), 180(T), 184(G), 189(C), 210(A), 216(T), 231(G), 257(T), 272(A), 280(C), 303(C), 312(A), 333(T), 354(G), 409(T), 588(G), 616(A), 627(G), 652(A), 672(G), 696(G), 735(C), 756(A), 795(A), 819(G) |
| *C. rotundata* | 48(C), 105(C), 180(C), 184(T), 189(A), 210(G), 216(C), 231(A), 257(G), 272(G), 280(T), 303(T), 312(G), 333(G), 354(A), 409(A), 588(A), 616(C), 627(A), 652(G), 672(A), 696(A), 735(A), 756(G), 795(C), 819(A) |
| *E. acoroides* | 30(C), 54(T), 105(T), 126(G), 129(T), 133(T), 143(T), 144(C), 145(C), 161(A), 165(G), 173(C), 199(T), 204(G), 239(T), 332(T), 352(T), 372(T), 510(A), 582(A), 583(G), 604(A), 623(C), 671(C), 696(A), 724(T), 728(C) |
| *T. hemprichii* | 33(T), 114(C), 155(C), 189(T), 300(T), 337(T), 374(A), 409(A), 411(G), 451(T), 464(A), 593(T) |
| *H. beccarii (incl. H.* spec. A**)* | 18(C), 66(G), 162(T), 208(A), 289(C), 296(A), 395(T), 498(A), 500(G), 513(G), 554(A), 557(T), 599(T), 712(C), 722(C), 743(C), 818(A), 831(A) |
| *H. ovata (incl. H. ovalis*, H*. spec. B**)* | 207(A), 342(T), 596(A), 605(A), 643(A) |
| *H. ovalis (incl. H. decipiens)* | 836(T), 846(G), 852(A), 855(A), 858(A) |
| *H. ovalis* subsp. *ramamurthiana* | 836(C), 846(C), 852(G), 855(T), 858(G) |
| *H. stipulacea* | 346(A), 399(G), 486(A), 641(G) |
| *H. pinifolia** | 14(G), 827(G) |
| *H.* spec*.** | 618(C), 624(C), 798(G), 846(C), 852(G), 854(C), 855(T), 858(G) |
| *H. uninervis (incl. H. pinifolia)* | 12(T) |
| *H. wrightii* | 289(A), 601(C) |
| *S. isoetifolium* | 289(G), 601(A) |
| *Z. marina (incl. all locations)* | 12(A), 63(T), 84(A), 118(A), 135(A), 143(T), 144(A), 156(G), 180(T), 192(T), 222(T), 227(C), 240(G), 247(A), 251(T), 254(G), 265(T), 273(A), 289(G), 297(G), 310(T), 318(A), 331(A), 346(A), 352(C), 355(G), 395(T), 413(G), 507(C), 513(A), 522(A), 536(G), 560(A), 564(T), 580(C), 625(A), 654(C), 681(A), 682(A), 686(A), 699(C), 724(T), 740(G), 742(T), 744(C), 765(A), 819(T) |
| *Z. noltii (incl. all locations)* | 12(G), 63(C), 84(G), 118(G), 135(G), 143(C), 144(G), 156(A), 180(C), 192(C), 222(A), 227(T), 240(A), 247(G), 251(C), 254(T), 265(G), 273(G), 289(A), 297(A), 310(C), 318(G), 331(T), 346(G), 352(G), 355(T), 395(G), 413(C), 507(T), 513(C), 522(G), 536(A), 560(G), 564(C), 580(T), 625(G), 654(T), 681(G), 682v686(G), 699(T), 724(G), 740(T), 742(G), 744(A), 765(G), 819(G) |
